# Novel arsenic-transforming bacteria and the diversity of their arsenic-related genes and enzymes arising from arsenic-polluted freshwater sediment

**DOI:** 10.1038/s41598-017-11548-8

**Published:** 2017-09-11

**Authors:** Maria L. S. Suhadolnik, Ana P. C. Salgado, Larissa L. S. Scholte, Lucas Bleicher, Patrícia S. Costa, Mariana P. Reis, Marcela F. Dias, Marcelo P. Ávila, Francisco A. R. Barbosa, Edmar Chartone-Souza, Andréa M. A. Nascimento

**Affiliations:** 1 Departamento de Biologia Geral, Instituto de Ciências Biológicas, Universidade Federal de Minas Gerais, Belo Horizonte, Minas Gerais Brazil; 2 Centro de Pesquisas René Rachou – FIOCRUZ, Belo Horizonte, Minas Gerais Brazil; 3 Departamento de Bioquímica e Imunologia, Instituto de Ciências Biológicas, Universidade Federal de Minas Gerais, Belo Horizonte, Minas Gerais Brazil

## Abstract

Bacteria are essential in arsenic cycling. However, few studies have addressed 16S rRNA and arsenic-related functional gene diversity in long-term arsenic-contaminated tropical sediment. Here, using culture-based, metagenomic and computational approaches, we describe the diversity of bacteria, genes and enzymes involved in AsIII and AsV transformation in freshwater sediment and in anaerobic AsIII- and AsV-enrichment cultures (ECs). The taxonomic profile reveals significant differences among the communities. *Arcobacter*, *Dechloromonas*, *Sedimentibacter* and *Clostridium thermopalmarium* were exclusively found in ECs, whereas *Anaerobacillus* was restricted to AsV-EC. Novel taxa that are both AsV-reducers and AsIII-oxidizers were identified: *Dechloromonas*, *Acidovorax facilis*, *A*. *delafieldii*, *Aquabacterium*, *Shewanella*, *C*. *thermopalmarium* and *Macellibacteroides fermentans*. Phylogenic discrepancies were revealed among the *aioA*, *arsC* and *arrA* genes and those of other species, indicating horizontal gene transfer. ArsC and AioA have sets of amino acids that can be used to assess their functional and structural integrity and familial subgroups. The positions required for AsV reduction are conserved, suggesting strong selective pressure for maintaining the functionality of ArsC. Altogether, these findings highlight the role of freshwater sediment bacteria in arsenic mobility, and the untapped diversity of dissimilatory arsenate-reducing and arsenate-resistant bacteria, which might contribute to arsenic toxicity in aquatic environments.

## Introduction

Arsenic (As), a toxic metalloid, is naturally found in Earth’s crust and is recognized by the World Health Organization as one of the ten chemicals of major public health concern^1^. This metalloid occurs in four oxidation states (+5, +3, 0 and −3), but in aquatic environments, the prevailing species are inorganic arsenate (AsV) and arsenite (AsIII), the latter of which is more toxic and mobile than AsV. Despite its low abundance, As is widespread in the environment; there, bacteria play an important role in its biogeochemical cycling, directly taking part in As speciation or doing so indirectly through redox interactions with other metals and nutrients, e.g., iron and nitrogen^2^. Nevertheless, such intricate ecological interactions remain to be investigated, particularly in anaerobic conditions^3^. As can also be introduced into the environment by anthropogenic sources, such as mining activity, smelting and metallurgy^4^. In Brazil, it is estimated that at least 390,000 tons of As have been released into the Iron Quadrangle, one of the world’s largest mining regions, since the beginning of its gold-mining activity in the 17^th^ century^5^.

Microorganisms have evolved a variety of mechanisms to cope with As toxicity; these mechanisms have direct implications on the speciation and mobility of As in the environment^6, 7^. As-transforming bacteria, both aerobes and anaerobes, are phylogenetically and physiologically diverse. The best-characterized genes involved in As metabolism include those of the *ars*, *aio* and *arr* operons, which can be found in both plasmids and chromosomes^8^.

The *ars* operon is a detoxification system that reduces AsV to AsIII, facilitating its cellular extrusion; microorganisms harboring this system are called arsenate-resistant microbes (ARMs). The *arr* operon is a respiratory system that leads to energy gain via dissimilatory reduction of AsV to AsIII in anaerobiosis; such microorganisms are referred to as dissimilatory arsenate-reducing prokaryotes (DARPs)^2^. The *aio* operon, formerly referred to as *aro*, is a detoxification system that promotes AsIII oxidation to AsV^7^. Moreover, an alternative AsIII-oxidation gene (*arxA*) has been detected under anaerobic conditions in the chemoautotrophic bacterium, *Alkalilimnicola ehrlichii* strain MLHE1, which can use AsIII as an electron donor to reduce nitrate^9^. ARMs and DARPs are of special concern in aquatic reducing environments, such as groundwater and sediments, because AsV reduction mobilizes the more toxic AsIII into the aqueous phase^2^.

The *asrC* gene of the *ars* operon codes for an arsenate reductase protein that can be structurally diverse; in prokaryotes, the best-characterized family is that of the *Escherichia coli* R773 plasmid, which uses reduced glutathione (GSH) to convert AsV to AsIII and possesses a cysteine residue in the catalytic site^10^. Respiratory arsenate reductase and arsenite oxidase are heterodimers that possess a large molybdopterin-containing catalytic subunit (encoded by *arrA* and *aioA* genes, respectively). Besides a molybdopterin center, ArrA also harbors a [4Fe-4S] cluster, while AioA of *Alcaligenes faecalis* contains a [3Fe-4S] cluster. Furthermore, Arr’s smaller subunit, ArrB, contains three to four [4Fe-4S] clusters, while Aio’s smaller subunit contains a Rieske-type [2Fe-2S] cluster^11, 12^. They belong to the dimethyl sulfoxide reductase (DMSO) family of molybdenum enzymes, forming distinct phylogenetic clades^9^.

Investigations of the bacterial community structure in As-rich natural environments, using traditional isolation techniques and different metagenomics 16S rDNA-targeted molecular approaches, have been reported^13–16^. Although metagenomics provides comprehensive information about bacterial community structure, it does not yield direct evidence of the relationship between these organisms and the functions that they play in the environment, in contrast to culture-based techniques; however, using only these latter techniques cannot provide a broad picture of the structure, functionality and phylogeny of the proteins encoded by the genes under scrutiny.

In this context, combining anaerobic As-enrichment culturing, 16S rRNA and As-related functional gene approaches, and a computational analysis of As-metabolizing protein sequences would allow a thorough investigation of the anaerobic bacteria and As-related gene diversity. For this purpose, we identified bacteria from both anaerobic AsIII- and AsV-enrichment cultures (EC) containing As-contaminated sediment and the whole bacterial community of this sediment, using 16S rRNA next generation sequencing. Moreover, the functional genes related to As cycling were evaluated, including those for arsenite oxidase (*aioA*) and arsenate reductases (*arsC* and *arrA*). Computational analyses of the ArsC and AioA enzymes were performed to detect functional and structurally important positions in the translated sequences obtained in this study and from Pfam database by analyzing the context of their homolog families.

## Results

### Environmental parameters

The metal concentrations and physicochemical characteristics of the samples from the Mina Stream are summarized in Table 1. Fe, Cu, As, Zn, Al and Mn were the metals present in the highest concentrations in the sediment sample. Cu, Zn and As concentrations exceeded the limits established by the Brazilian environmental regulations (Conselho Nacional do Meio Ambiente – CONAMA)^17^ for sediment. Mina Stream can be characterized as a mesothermal (19.8 °C) oxidized environment with highly oxygenated (71.4% oxygen saturation) and circumneutral waters (pH = 6.8). Nitrogen and phosphorus ratio was greater than 9, which indicates that phosphorus is the most limiting nutrient. Thus, the trophic status of the water was eutrophic during the sampling period, a classification based on total phosphorous levels according to the model of Salas & Martino^18^.

**Table 1:** Metal concentrations in sediment, physicochemical characteristics of Mina Stream water, and their limits as permitted by Brazilian law (Conselho Nacional do Meio Ambiente – CONAMA).

Metals	Sediment (mg Kg^−1^)	CONAMA (mg Kg^−1^)	Physicochemical Parameters	Water	CONAMA
Fe	1811.6	NE^*^	Temperature (°C)	19.8	NE
Ni	5.8	18	pH	6.8	6–9
Mn	327.5	NE	Dissolved Oxygen (mg L^−1^)	6.3	>5
Cu	206.3	35.7	Oxygen Saturation (%)	71.4	NE
Pb	5.2	35	Conductivity (µs cm^−1^)	1500	NE
Zn	43.4	123	NO_2_^−^ (µg L^−1^)	7.4	≤100
Al	543.4	NE	NO_3_^−^ (µg L^−1^)	20	≤1000
Cr	1.6	37.3	NH_4_^+^ (µg L^−1^)	644.776	≤3700
As	42.9	5.9	Total P (µg L^−1^)	40.2	≤30
Hg	<0.1	0.17	PO_4_^3−^ (µg L^−1^)	5.25	NE
Mg	164.2	NE			

*NE- Not established by CONAMA.

### Community diversity overview

**Figure 1:**
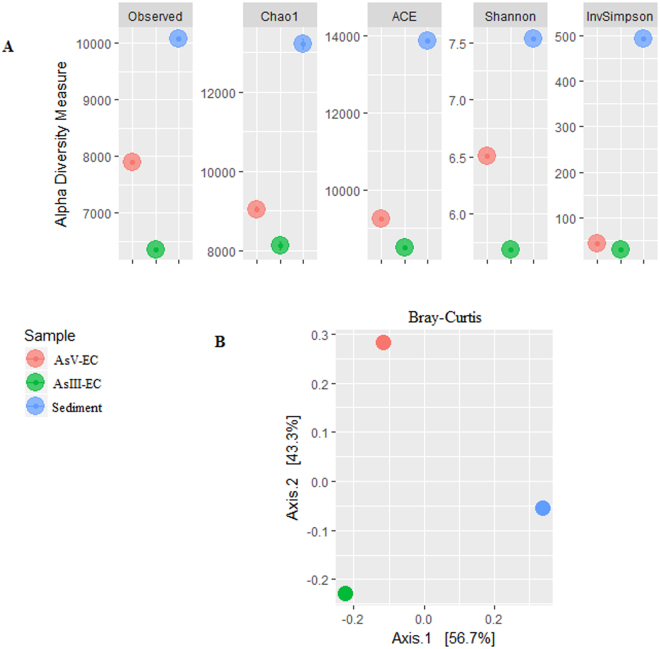
Diversity indexes. (**A**) Alpha diversity of bacterial communities in AsV- and AsIII-enrichment cultures (EC) and sediment. (**B**) PCoA plot of the bacterial community structure based on Bray-Curtis distances.

The community of the whole sediment showed higher diversity compared to both ECs (Fig. 1A), and the AsV-EC community was more diverse than that of AsIII-EC. EC communities were dominated by fewer operational taxonomic units (OTUs), opposed the case observed in the whole sediment community, as revealed by an inverse Simpson’s index analysis. The beta diversity result clearly showed dissimilarity among the communities (Fig. 1B).

### Microbiota composition

**Figure 2:**
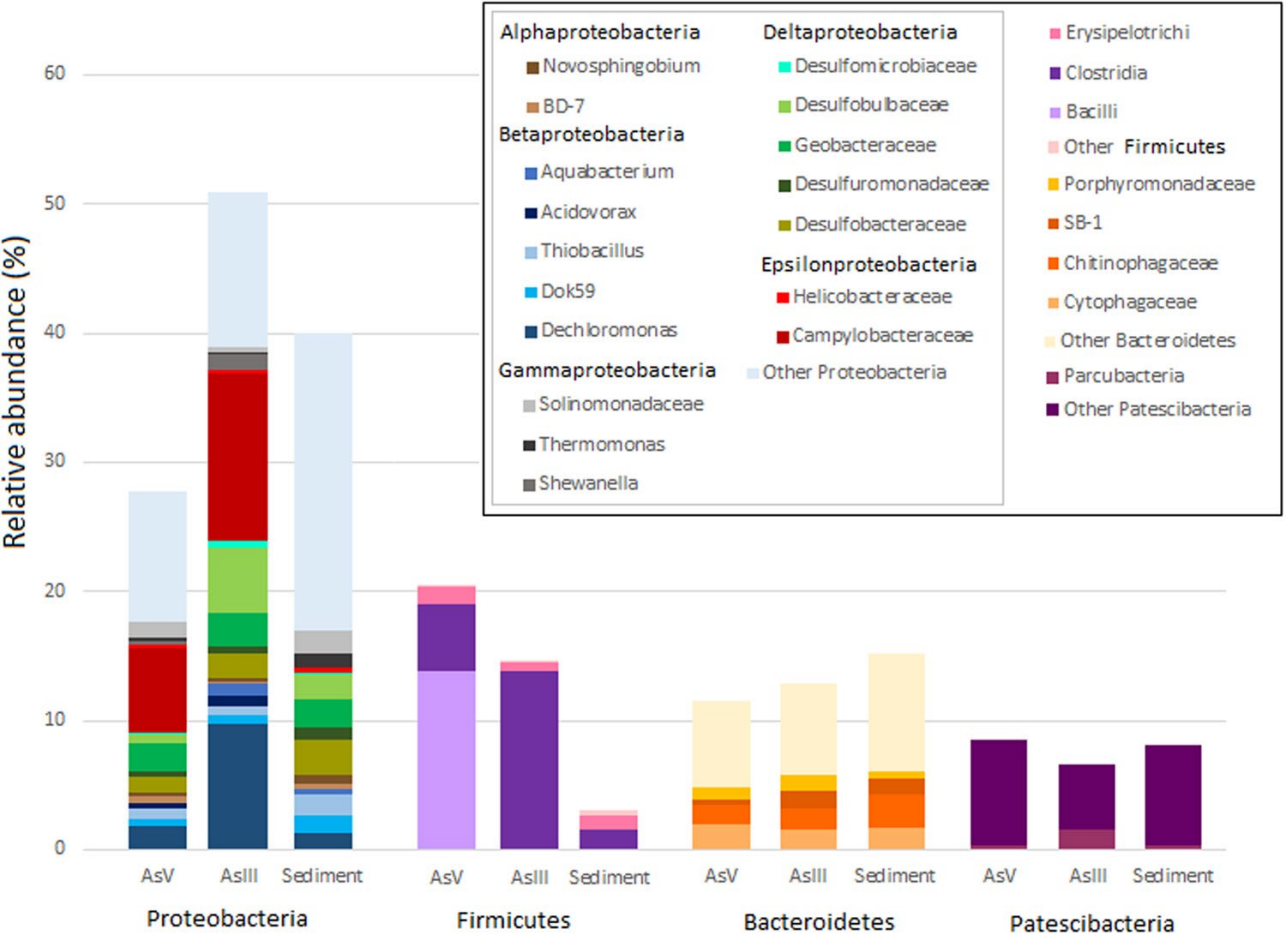
Taxonomic profile. The taxonomic distribution of the most abundant phyla observed in AsV- and AsIII-enrichment cultures (EC) and sediment. Taxa with relative abundance <1% in all samples were grouped as “Other”.

In total, 497,949 high-quality reads (mean of 165,983 per sample) were grouped into 19,708 OTUs (9,254 in AsV-EC, 8,654 in AsIII-EC, and 14,236 in sediment) distributed across 54 phyla. The number of reads in each sample and the OTUs’ taxonomic assignments are summarized in Table S1. The dominant phyla were Proteobacteria, Firmicutes, Bacteroidetes and Patescibacteria (Fig. 2).

Proteobacteria was the most abundant phylum, and Betaproteobacteria, the predominant class, had a relatively even distribution (34.5% in sediment, 28.6% in AsV-EC and 34.4% in AsIII-EC). *Dechloromonas*-related reads were noticeably more abundant in the AsIII-EC (55.7% of all betaproteobacterial reads) than in AsV-EC and in sediment (23.6 and 9.7%, respectively). *Dok59*, one of the most abundant genera in sediment (9.5% of all betaproteobacterial reads), was also detected as a minor fraction in the ECs (7.1% in AsV-EC and 4.1% in AsIII-EC). Other betaproteobacterial groups favored by anaerobic arsenic enrichment conditions included *Acidovorax*, represented by the species *A*. *facilis*, *A*. *delafieldii* and *Aquabacterium*. Furthermore, it is noteworthy that one unique *Thiobacillus*-related OTU (OTU 7) was the most abundant in AsV-EC and in sediment (9.7 and 11.7% of all the betaproteobacterial OTUs, respectively).

Deltaproteobacteria were well represented in all three samples (30.6% in sediment, 23.7% in AsV-EC and 25.5% in AsIII-EC), mainly by Desulfobacteraceae, Desulfobulbaceae, Desulfomicrobiaceae, and Geobacteraceae families. A high proportion of unclassified reads within Desulfobacteraceae (70 to 85.2%) was observed. In Desulfobulbaceae, most reads belonged to the *Desulfobulbus* genus (27.1% in AsV-EC, 86.2% in AsIII-EC, and 17.8% in sediment). The family Desulfomicrobiaceae was represented by a single genus, *Desulfomicrobium*. All the reads of Geobacteraceae were affiliated with the Fe(III)-reducing bacterium, *Geobacter*.

Alphaproteobacteria abundance decreased in the enrichment cultures (9.3% in AsV-EC and 3.7% in AsIII-EC) compared to the sediment (14.7%). Order BD7-3 and the family Sphingomonadaceae (genus *Novosphingobium*) were particularly abundant.

Epsilonbacteria reads were predominantly affiliated with Campylobacteraceae in both ECs’ communities, particularly with the *Arcobacter* genus. Within Gammaproteobacteria, two out of three dominant groups were classified at the genus level as *Shewanella* and *Thermomonas*. *Shewanella* reads were almost absent in sediment (0.03% of all gammaproteobacterial reads), but significantly increased in AsV-EC (8.9%) and AsIII-EC (24.2%). In contrast, *Thermomonas* was found in lower proportions in the ECs (8.1% in AsIII-EC, 3.7% in AsV-EC, 13.8% in sediment, of all gammaproteobacterial reads). Another abundant OTU was affiliated with the family Sinobacteraceae (39.1% in AsV-EC, 7.3% in AsIII-EC and 24% of all Gammaproteobacteria reads), which are now known as the Solimonadaceae^19^.

The relative abundances of dominant classes for Firmicutes differed significantly between AsV-EC and AsIII-EC/sediment communities. Bacilli and Clostridia classes became notably enriched in AsV-EC and AsIII-EC, respectively, compared to the sediment. The genus *Anaerobacillus* (63.5% of all Firmicutes reads) was specific from AsV-EC, whereas *Clostridium thermopalmarium* (71% of all Firmicutes reads in a single OTU) predominated in AsIII-EC. Other genera that reached >1% abundance were *Lactobacillus* (2.5%) and *Desulfosporosinus meridiei* (1.1%) in AsV-EC (poorly represented in AsIII-EC) and *Sedimentibacter*, which was equally represented in AsV-EC and AsIII-EC (2.5%).

Among the dominant phyla, Bacteroidetes presented the highest proportion of unclassified reads. Interestingly, the most abundant Bacteroidetes OTUs (OTU 05: 10.5% in AsV-EC, 6.7% in AsIII-EC and 5.5% in sediment) remain unassigned to any class. Moreover, reads belonging to families Cytophagaceae, Chitinophagaceae and SB-1 were unclassified at the genus level. Within Porphyromonadaceae, the strictly anaerobic genus *Paludibacter* ^20^ accounted for 1% of all reads of AsIII-EC.

Finally, the phyla Parcubacteria, Gracilibacteria and Microgenomates (formerly referred to as OD1, GN02 and OP11, respectively) form the Patescibacteria superphylum, which was proposed by Rinke *et al*.^21^ and accounted for 8.3% of AsV-EC, 5.2% of AsIII-EC and 7.9% of sediment microbes.

### Molecular diversity of the *aioA*, *arrA* and *arsC* genes

**Figure 3:**
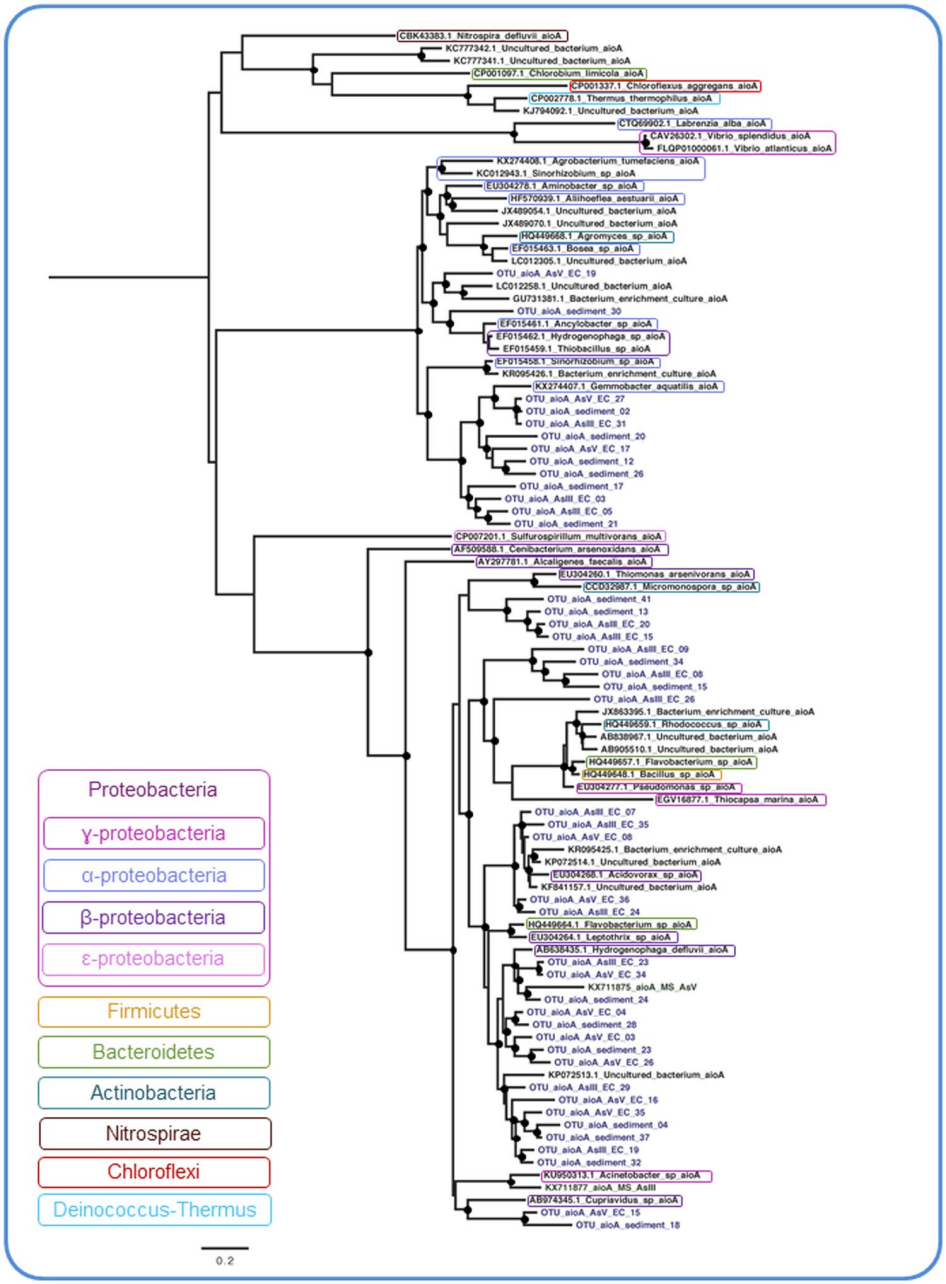
The evolutionary relationships of *aioA* sequences. The phylogeny was reconstructed by maximum likelihood with 96 nucleotide sequences, 541 sites and TVM + I + G + F selected as best fit model. Support values for each node were estimated using the Akaike Likelihood Ratio Test (aLRT). Nodes with support values higher than 70% are highlighted with black circles. The reference sequences retrieved from the non-redundant database of the NCBI are shown in black and operational taxonomic unitis (OTUs) from AsV- and AsIII-enrichment cultures (EC) and sediment are shown in blue.

**Figure 4:**
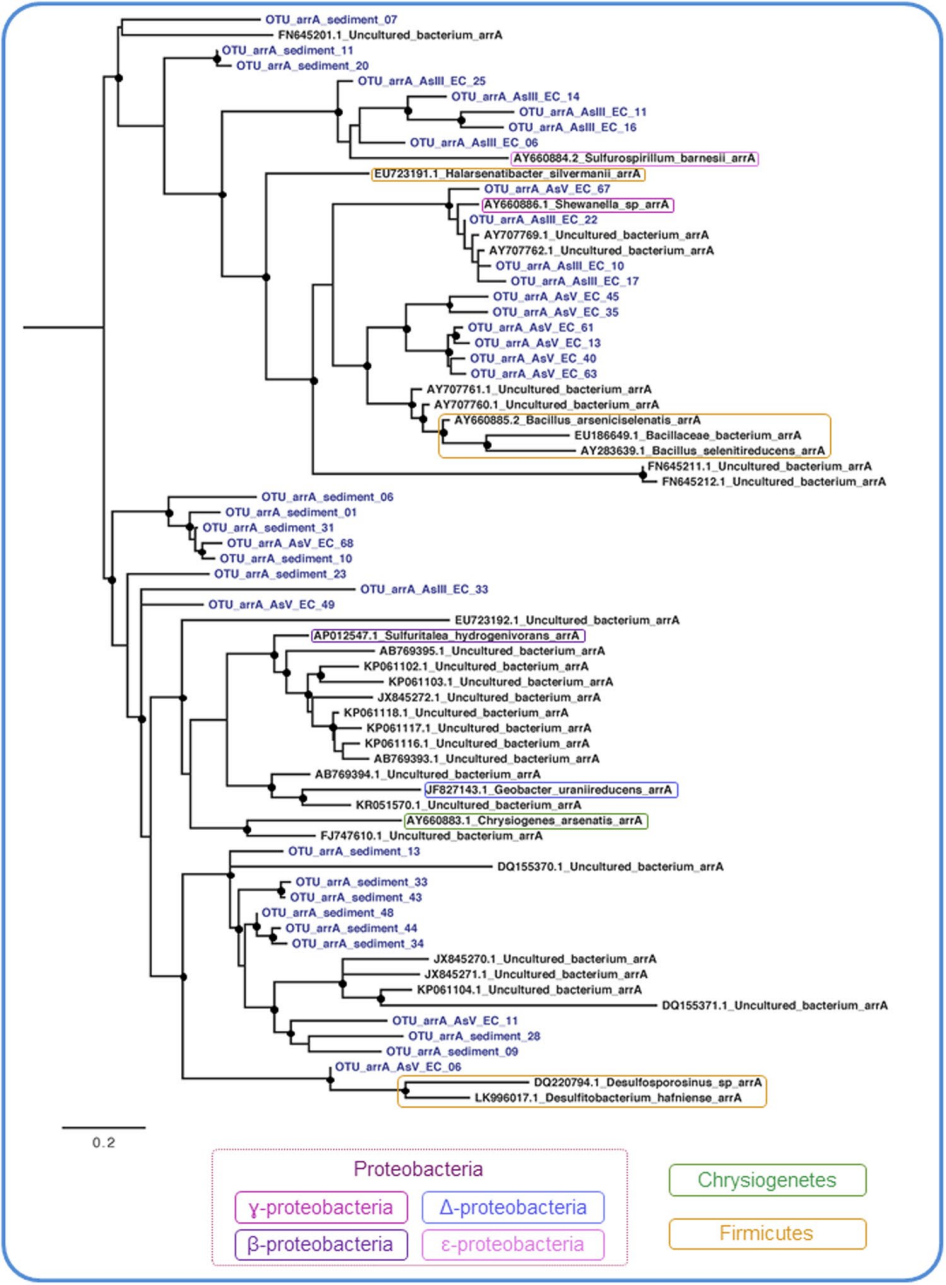
The evolutionary relationship of *arrA* sequences. The phylogeny was reconstructed by maximum likelihood with 71 nucleotide sequences, 160 sites and TIM3 + I + G + F selected as best fit model. Support values for each node were estimated using the Akaike Likelihood Ratio Test (aLRT). Nodes with support values higher than 70% are highlighted with black circles. The reference sequences retrieved from the non-redundant database of the NCBI are shown in black and operational taxonomic unitis (OTUs) from AsV- and AsIII-enrichment cultures (EC) and sediment are shown in blue.

**Figure 5:**
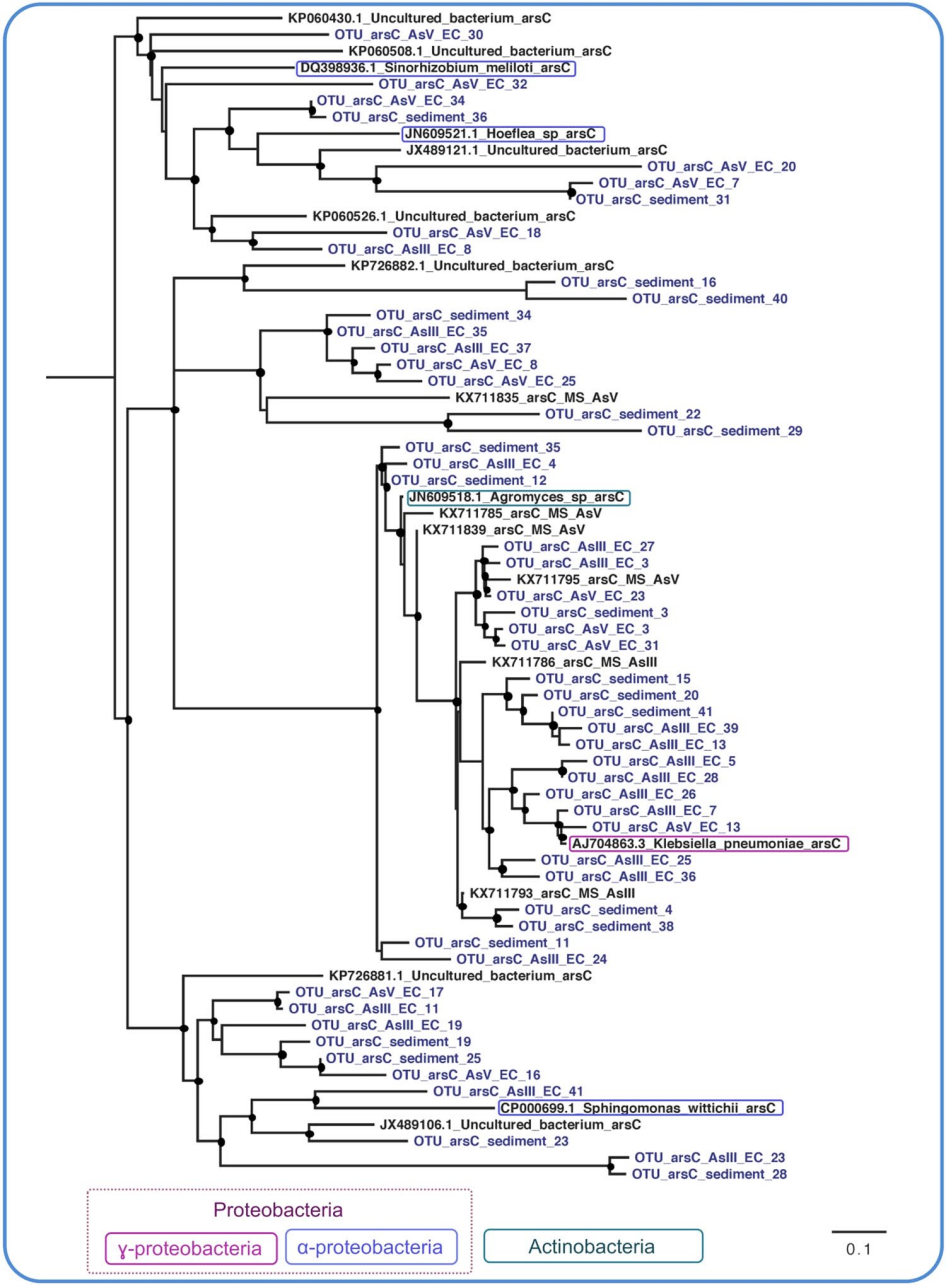
The evolutionary relationship of *arsC* sequences. The phylogeny was reconstructed by maximum likelihood with 71 nucleotide sequences, 352 sites and TIM2 + I + G + F selected as best fit model. Support values for each node were estimated using the Akaike Likelihood Ratio Test (aLRT). Nodes with support values higher than 70% are highlighted with black circles. The reference sequences retrieved from the non-redundant database of the NCBI are shown in black and operational taxonomic unitis (OTUs) from AsV- and AsIII-enrichment cultures (EC) and sediment are shown in blue.

Coverage values of the AsIII-EC libraries (68%, 70% and 62%, respectively for *arsC*, *arrA*, and *aioA*), AsV- EC libraries (70%, 83% and 64%) and sediment libraries (60%, 71% and 61%) indicated that most of the diversity of these genes was detected. To provide insights into the evolutionary relationships among the *arrA*, *arsC* and *aioA* OTUs and reference homologs, phylogenetic reconstructions were made. They revealed high genetic divergence from the sequences in databases (Figs 3, 4 and 5) and clear discrepancies between these genes and the phylogenies of known species. An evolutionary analysis revealed low similarity among *arrA* sequences obtained from AsV-EC, AsIII-EC and sediment, as well as between them and reference sequences. In contrast, *arsC* OTUs from different samples were scattered in all the clusters. In total, *aioA* OTUs segregated into three different clusters.

### Computational analysis of ArsC and AioA

**Figure 6:**
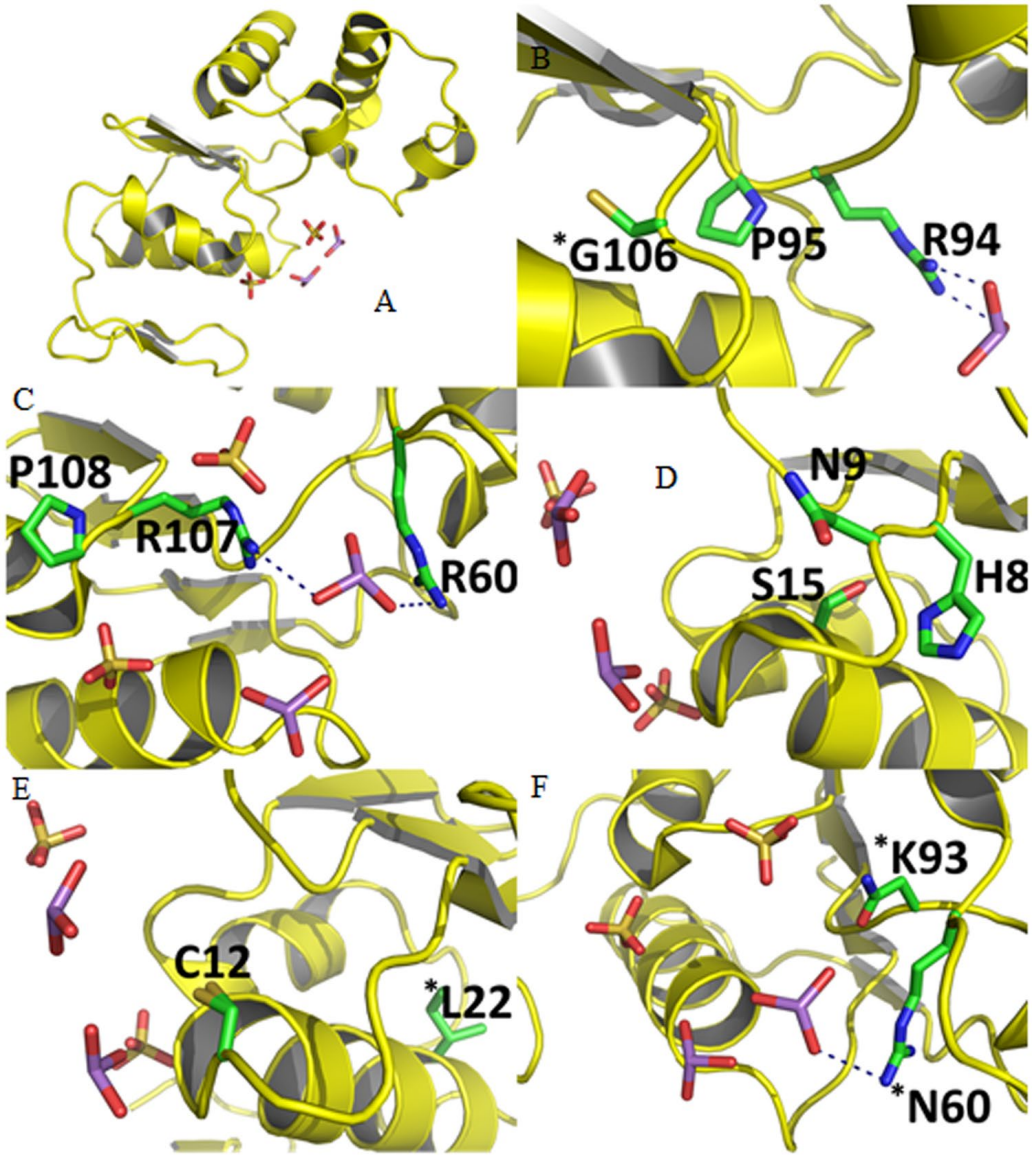
ArsC enzyme from *E*. *coli*. (**A**) Overview of ArsC from *E*. *coli* bound to arsenate (represented as sticks in purple and red) and sulfate (yellow and red). (**B**) Three most conserved positions in ArsC homologs. (**C**–**F**) The four sets of coevolved positions among ArsC homologs. Asterisks refer to positions that show an alternative residue in ArsC from *E*. *coli*.

Multiple sequence alignment analysis of the ArsC domain PF03960 (Fig. 6A), belonging to glutathione-reducing ArsC family previously described, showed only three highly conserved positions (>80%) (Fig. 6B), which were located near the arsenate binding site Cys12, and four sets of coevolved positions (Fig. 6C–F), summarized in Table 2 and Fig. 6. This ArsC domain is shared not only by arsenate reductases but also by the regulatory proteins Spx and mgsR^22^; it is noteworthy that the catalytic cysteine (Cys12) is not conserved across all homologs, being present in only 54% of the sequences.

**Table 2:** Highly conserved residues and sets of coevolved positions in ArsC domain PF03960 identified by multiple sequence analysis.

	Residues	Functional or structural role	Reference	% Residue’s conservation among the 2239 Pfam filtered sequences	% Residue’s conservation among translated sequences obtained in this study
Highly conserved residues	Cys12	Required for arsenic resistance: catalytic residue	88	53	NIA
	Arg94	Required for arsenic resistance	10	93	100
	Pro95	Precedes a beta-strand			100
	Gly106 or Cys106	Gly106 is also conserved in this family’s Spx regulatory protein			94 (Cys106)
Coevolved positions Set 1	Arg60	Required for arsenic resistance	10	33	100
	Arg107	Required for arsenic resistance	10		
	Pro108	Starts an alpha-helix			
Coevolved positions Set 2	His8	Required for arsenic resistance: lowers Cys pKa	89	33	NIA
	Asn9	Related to positioning of catalytic Cys	90		NIA
	Ser15	Stabilization of the active site loop	10		NIA
Coevolved positions Set 3	Leu22 or Ile22			45 (Leu22)	NIA
	Cys12	Catalytic residue			
Coevolved positions Set 4	Lys93	Found in ArsC’s paralogues YffB from *E*. *coli* and YusI from *Bacillus subtilis*.	23, 91	50	0
	Asn60				

NIA: not included in amplified region.

It is remarkable that most residues related to the arsenate reductase function, instead of only the strictly conserved residues, are present in the coevolved sets, thus making them useful tools to distinguish potential ArsC sequences from its homologs that have different functions. Sets 1 (Arg60, Arg107, Pro108) (Fig. 6C) and 2 (His8, Asn9 and Ser15) (Fig. 6D) are required for arsenic resistance, whereas set 3 (Fig. 6E) may be present in Spx regulatory proteins, and set 4 (Fig. 6F) is characteristic of the ArsC paralog proteins YffB from *E*. *coli* and YusI from *Bacillus subtilis*. The former can function as a glutathione-dependent thiol reductases^23, 24^. Spx proteins can be segregated from ArsC because they possess the highly conserved Gly106 (Gly104 for *Bacillus subtilis* Spx), while Cys106 is more prevalent in ArsC; Spx proteins also lack residues in the coevolved sets 1, 2 and 4. Moreover, in ArsCs such as that of *E*. *coli*, isoleucine is more commonly found at position 22.

**Figure 7:**
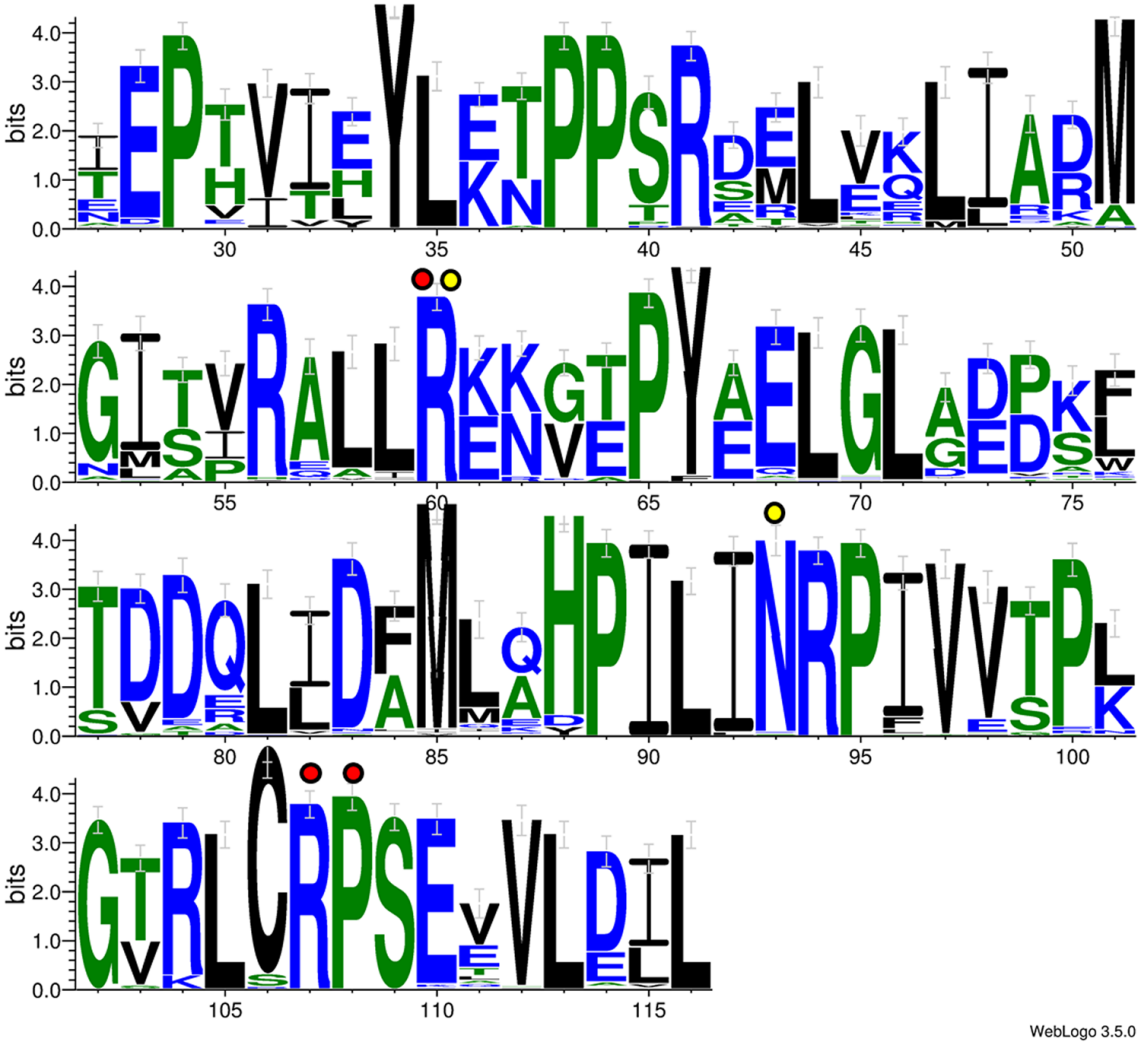
Residue-type distribution of the translated amplicon from putative ArsCs. The numbering used corresponds to ArsC protein from *E*. *coli*. Error bars indicate a Bayesian confidence level of 95%. The positions from the two coevolving sets detected in the translated amplicon, 1 (R60, R107 and P108) and 4 (N60 and K93) are indicated in red and yellow circles, respectively.

Translation of the sequences amplified with the *arsC* primers yield residue sequences spanning the region from positions 27 to 116 in *E*. *coli* ArsC. Therefore, many positions found to be important for structure and function and putatively determining subclass adherence are included in the amplified sequences. As shown in Table 2, Arg94 and Pro95 are present in all sequences, but position 106 is dominated by Cys (94%); as previously stated, glycines are more common in other members of the family. The coevolved sets, which are more likely to identify subclasses in the alignment, follow the previously described trend for ArsC-related residues. The overall distribution of residues in the translated amplicon (Fig. 7) shows that coevolved set 1 is present in all of our sequences, and none of them possess coevolved set 4, discarding the paralogs YffB and YusI.

The molybdopterin oxidoreductase domain (PF00384) analysis revealed thirteen highly conserved (>80%) residues and six sets of coevolved residues. The conserved residues are Arg112, Pro116, Trp167, Ala170, Gly258, Pro287, Asp307, Ala518, Gly557, Gly563, Leu778, Pro779, and Glu785. The two AioAs in the SwissProt database (Q7SIF4, from *Alcaligenes faecalis* and Q8GGJ6, from *Herminiimonas arsenicoxydans*) conserve all those residues except the Gly at position 557 (it is a His in Q7SIF4 and a Gln in Q8GGJ6). This domain is present in a large number of different proteins that catalyze oxidation-reduction reactions, and one of the many proteins with this domain (reviewed in^25^) is the 75-kDa subunit of the NADH-ubiquinone oxidoreductase, which does not conserve residues Pro287, Asp307, Ala518, Gly557 and Gly563. Ala518 is always absent, while the other positions have other residues with no preference except that for apolar residues (mostly Leu) in the Asp307 position. The alignment is also populated by many NapAs, periplasmic nitrate reductases, which usually have a threonine instead of P287. Finally, NADH-quinone oxidoreductase subunit G (NUOG) usually lacks most of those residues except for the last three.

The six sets of coevolved residues are Pro304, Ala774, Asp775, Leu778, Pro779 and Glu785 (the two AioAs at SwissProt have all residues except for the Asp at position 775, with a His instead); Pro287, Asp307, Gly563 and Gly557 (the last Gly is not present in AioAs); Arg112 and Pro116 (present in all sequences except for most NUOGs, the two tetrathionate reductase A subunits and a few other members with a single sequence at SwissProt); Ile202 and Thr266; Asn260 and Gly535 and finally Gly560 and Gly565.

**Figure 8:**
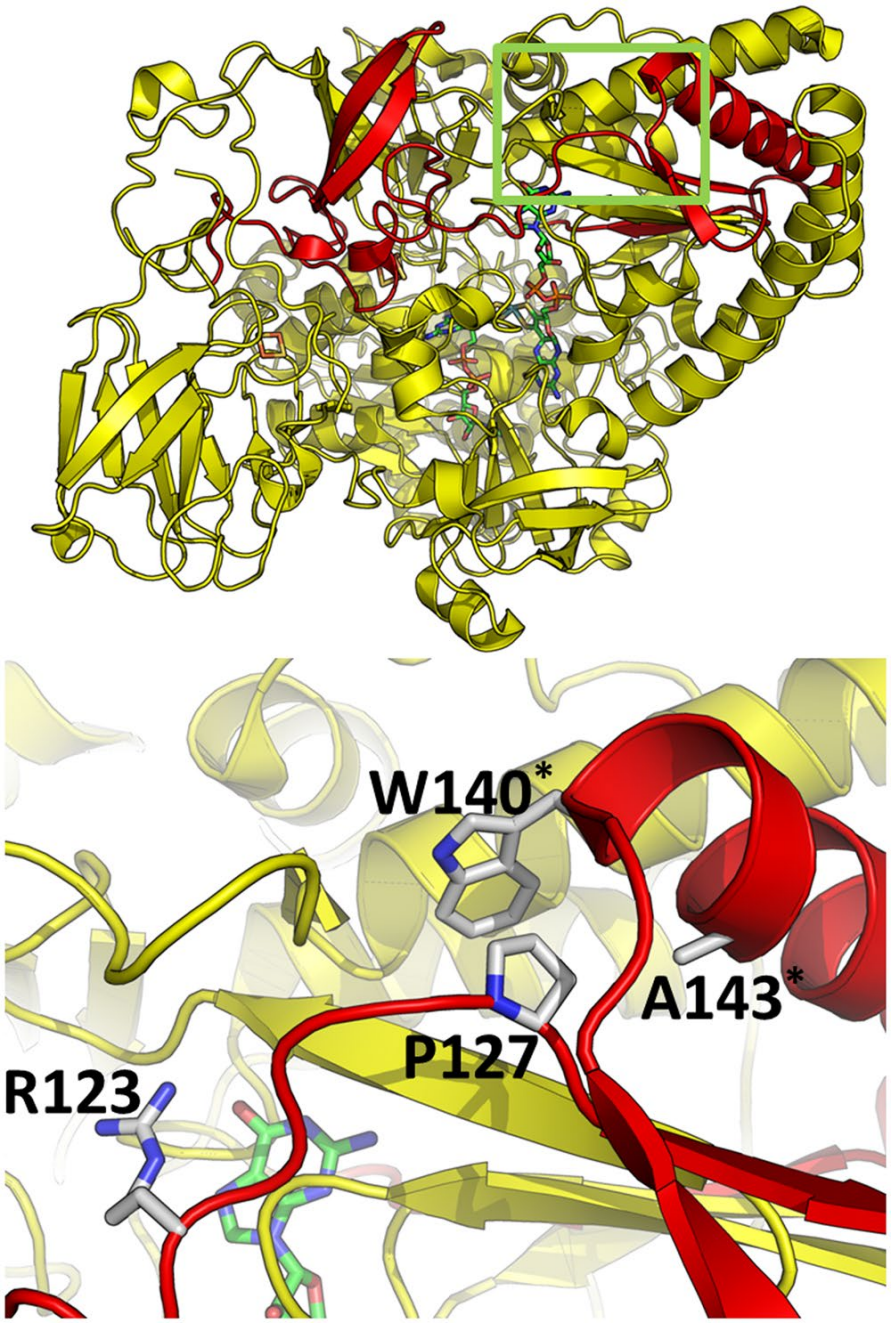
Three-dimensional structure of the arsenite oxidase protein complex from *Rhizobium* species strain NT-26, complexed to molybdopterin guanosine dinucleotide (MGD). The region shown in red corresponds to the translation of amplified sequences in this study. The rectangle in green, zoomed in the bottom panel, shows the region containing the four conserved residues codified in the amplicons. The residues marked with an asterisk correspond to those not present in the translated amplicons found in this study (position 140 is dominated by glutamate, while position 143 is dominated by tryptophan). The ligand in the background, with carbon atoms shown in green, corresponds to MGD.

The *aioA* amplicons obtained in this study were translated into a sequence which we mapped to a typical structure in this protein family – the arsenite oxidase protein complex from *Rhizobium* strain NT-26 (PDB: 4AAY). The amplified region corresponds to residues 64–185 in chain A. This encompasses only the first four highly conserved residues among the molybdopterin domains, which in AioA is contained in the catalytic subunit, but is also present in most members in SwissProt, except for NUOGs and tetrathionate reductase A subunits (TTRAs). In the structure, they are to Arg123, Pro127, Trp140 and Ala143. For the sequences found in this study, only the first two highly conserved residues (Arg112 and Pro116) are present; positions 167 and 170 are dominated by glutamate and tryptophan, respectively. As opposed to ArsCs, the coevolved positions are virtually all outside the translated amplified region, except for Arg112 and Pro116 (Arg123 and Pro127 in the structure). As stated in the previous session, these two residues are common in most types of proteins containing a molybdopterin domain, the single significant exception being the NUOGs, in which these residues are commonly absent. The location of the amplified regions and the four conserved residues, as mapped in the structure, is shown in Fig. 8.

## Discussion

In a previous study, our group identified aerobic As-transforming bacterial isolates in Mina Stream’s sediment, and characterized the microbial community’s As-related genes through metagenomic analysis^16^. Here, we have broadened those findings by combining molecular and computational approaches to obtain a comprehensive insight into anaerobic bacterial taxonomy and their arsenic-related genes and enzymes diversity, which are of special environmental concern. Such approaches revealed an unprecedent diversity of bacteria acting as AsIII oxidizers and/or AsV reducers, many described here for the first time; and naturally occurring mutations of *arsC* and *aioA* genes.

The diversity analysis indicates that the whole sediment community is more diverse than that of the ECs, which was expected; the whole sediment harbors the actual community, whereas the ECs favored only the growth of As-transforming bacteria. The AsV-EC community was more diverse than that of the AsIII-EC, very likely due to the high toxicity of AsIII. The dominance by fewer OTUs that was observed in the EC communities, as revealed by the inverse Simpson index, suggests that they are shaped by competitive interactions, i.e., only a few species that are better adapted to the conditions prevail^26^.

Proteobacteria, Firmicutes, Bacteroidetes and Patescibacteria dominated all samples. This result is consistent with the data from As-rich environments from which these phyla have been recovered^15, 27^. Overall, there was a variation in the dominant OTU abundance across samples; some of them were absent or rare in the other samples, which could be explained as an effect of the anaerobic enrichment process. The presence of many shared taxa in both ECs suggests that different As-resistance and metabolism-related gene clusters can coexist in a single genome^8^, conferring to the same bacterium the ability to both reduce and oxidize As, thus increasing their fitness in As-rich environments. Moreover, this scenario implies that AsV-respiring bacteria can also have the detoxifying arsenate reductase, encoded by *arsC*, as is the case for *Shewanella* sp. strain ANA-3^28^. Hence, bacteria found in AsV-EC could be DARPs, ARMs, or both.

DARPs are more concerning than ARMs because they can reduce both sorbed and aqueous AsV; this behavior is probably because ArrA is a periplasmic enzyme and ArsC is cytoplasmic, thereby transforming only dissolved AsV inside the cell^2^. AsV-respiring genera such as *Clostridium*, *Bacillus*, *Desulfosporosinus*, *Shewanella*, *Sulfurospirillum* and *Desulfomicrobium* ^2^ were identified in this study, suggesting that intense AsV-reducing activity may be taking place in the sediment of the Mina Stream. DARPs can use a wide array of electron acceptors besides AsV, such as nitrate, sulfur and iron species; according to Oremland & Stolz^6^, this versatility may be an important ecological factor because these elements interact with As in the environment. The present study supports this view, as iron was found in very high concentration (1811.6 mg/kg) in the sediment sample.

Within the class Betaproteobacteria, *Dechloromonas*-related OTUs were remarkably more abundant in the AsIII-EC than in the other samples, suggesting that this genus might contain members with higher fitness in AsIII-rich anaerobic conditions. Indeed, this finding agrees with Sun *et al*.^29^, who reported that the anaerobic oxidation of AsIII is linked to chlorate reduction by members of *Dechloromonas*. *Dok59*, detected in the ECs, has not been associated with As or metal resistance so far. It is noteworthy that the most abundant OTU in AsV-EC and in sediment was *Thiobacillus*-related. This genus can resist As and high toxicity metals^30^, and a recent study reported it as the dominant genus in high-As sediments^31^.

Other betaproteobacterial taxa favored by the ECs’ conditions were *Acidovorax facilis*, *A*. *delafieldii*, and *Aquabacterium*. Representatives of *Acidovorax* have been found in soil, sediment and groundwater contaminated with As^16, 32, 33^ and are known for their ability to oxidize AsIII^16^ and reduce nitrate^34^. Furthermore, *Acidovorax* strains isolated from gold mine soil and in the same sampling site of the present study have been reported to harbor both *arsC* and *aioA* genes^16, 34^. Indeed, as highlighted in a review by Andres & Bertin^8^, genomic analyses from different taxa revealed that As resistance and metabolism-related genes can both occur in the same genome. Thus, our findings are consistent with these studies, and here, for the first time, we report that the species *A*. *facilis*, and *A*. *delafieldii* have the potential ability to reduce and oxidize arsenic.

Members of the genus *Aquabacterium* have been found in As-contaminated groundwater samples^35^ and harbor the *arsC* gene in their genome^36^. In the present study, *Aquabacterium*-related reads were three-fold more abundant in AsIII-EC than in AsV-EC, suggesting that it might have a role in As reduction and oxidation.

The alphaproteobacterial order BD7-3 and the family Sphingomonadaceae, genus *Novosphingobium*, were particularly abundant. Members of BD7-3 are rare and poorly characterized, and 16S rDNA sequences belonging to this group were first recovered from deep-sea sediment samples^37^. In contrast, Sphingomonadaceae can be found in a wide variety of environments, from rhizosphere to freshwater^38^, and *Novosphingobium* sequences have been recovered from As-rich groundwater sediment^39^ and the bacterial floc of a filtration pilot plant for As, Fe and Mn removal^40^.

The occurrence of *Desulfobulbus*, *Desulfomicrobium and Geobacter* (Deltaproteobacteria) in the present study is consistent with previous reports. *Desulfobulbus* and *Desulfomicrobium* are strictly anaerobic bacteria that use sulfate and other sulfur compounds as electron acceptors^41, 42^ and can also perform AsV reduction^43, 44^. Nevertheless, their occurrence in the AsIII-EC is surprising because it means that in addition an AsV-reducing apparatus, they must also be equipped with AsIII-oxidizing machinery. As demonstrated by Islam *et al*.^45^, *Geobacter* takes part in As mobilization in As-rich aquifers; although this ability is not enzymatically mediated, the microbes possess an adequate genetic apparatus. According to the authors, *G*. *sulfurreducens* reduces Fe(III), and the resulting Fe(II) phases capture As and act as sinks for the metalloid in sediments^45^.

Epsilonproteobacteria is recognized as an ecologically important group of bacteria that is globally ubiquitous in marine and terrestrial ecosystems, particularly in deep-sea hydrothermal environments^46^. *Arcobacter-*related OTUs were very abundant in both the AsV-EC and AsIII-EC communities, suggesting that the members of this genus can act as either arsenite oxidizers or arsenate reducers in anoxic conditions. A previous study showed that *Arcobacter* dominated the bacterial 16S rRNA clone libraries from As-rich shallow-sea hydrothermal sediment^47^. Minor genera included *Sulfuricurvum* and *Sulfurimonas* (Helicobacteraceae). *Sulfuricurvum* dominated high-As sediments^31^ and the genus contains a representative whose genome sequencing revealed genes for tolerance to heavy metals and As^48^. To date, *Sulfurimonas* has not been described in environments with As. Nevertheless, the genome of *S*. *denitrificans* presents several genes encoding heavy metal efflux transporters^49^. Our findings point not only to the dominance of Epsilonproteobacteria in deep-sea hydrothermal environments^46^ but also their participation in the flux of other elements in addition to sulfur.

Concerning Gammaproteobacteria, *Shewanella* and *Thermomonas* were well represented. The former is a facultative anaerobe that can reduce AsV under anoxic conditions^28^. Moreover, *Shewanella* sp. strain ANA-3^28^ and *Thermomonas*, described by our group as novel arsenic-transforming genus^16^, harbor both *ars* and *arr* operons. Importantly, our findings expand the understanding of *Shewanella* in its possible role in AsIII oxidation. Another abundant group was Solimonadaceae, whose members are found in soil and freshwater and have ability to decompose chemical pollutants. Our findings suggest that members of this family might be AsV reducers and AsIII oxidizers.

Regarding Firmicutes, the genus *Anaerobacillus* was found exclusively in AsV-EC, whereas the AsIII-EC community was enriched with *Clostridium thermopalmarium*. Previous works demonstrated that members of the genus *Anaerobacillus* are capable of anaerobic respiration with arsenate as a terminal electron acceptor^50^. In contrast, *C*. *thermopalmarium*, whose type strain was isolated from palm wine^51^, has not yet been described with regard to As transformation. *Desulfosporosinus meridiei* and *Sedimentibacter* reached an abundance >1%. Although the genome of *D*. *meridiei* DSM 13257 harbors the *arsC* gene^52^, this species has not been implicated in As transformations in the environment so far. *Sedimentibacter* seems to be capable of both AsV reduction and AsIII oxidation, as observed in enrichment cultures^53^.

Within Bacteroidetes, the families Cytophagaceae, Chitinophagaceae and SB-1 were abundant in this study, the latter being poorly characterized so far. Members of Cytophagaceae have been found to be associated with the rhizosphere in As-contaminated soils^54^, and the bacterial strain SM-1 mediates the methylation and volatilization of As in paddy soils^55^. In contrast, Chitinophagaceae are chitinolytic organisms that degrade complex polysaccharides^56^ and are so far not associated with As metabolism. Other genera detected in this study were *Paludibacter* and *Macellibacteroides*, with a single species, *M*. *fermentas*. Gronow *et al*.^57^ identified in the genome of *Paludibacter* an arsenate reductase-like gene. Importantly, this report is the first that describes *Macellibacteroides* being recovered from As-contaminated sediment and possessing As-oxidizing and As-reducing activities.

Lastly, the Patescibacteria superphylum, characterized by having reduced metabolic capacities, is also involved in hydrogen production, sulfur cycling and anaerobic methane oxidation^58, 59^. Our research group has also reported the presence of Patescibacteria in relatively high abundance in freshwater sediment with high As and metal content^27^.

The reconstructed phylogeny for the *aioA*, *arrA* and *arsC* genes revealed a great sequence diversity, demonstrated by their high genetic divergence from sequences in database. The unequivocal discrepancies between these genes and the species phylogenies indicate the occurrence of horizontal gene transfer, as previously observed in other studies^60–62^. The segregation of OTUs into different clusters, as noted in *aioA’*s phylogenetic tree, may reflect genetic divergence due to niche segregation or the existence of functional variants of the gene. Furthermore, *arrA*’s evolutionary analysis suggested that the OTUs may be potentially novel functional variants once its tree distinctly showed low genetic similarity among sequences obtained from the three samples, as well as among reference sequences. Finally, *arsC* is known to be widespread, horizontally transferred and to have an evolutionarily conserved mechanism of detoxification^8^. Such characteristics were evident in its phylogenetic tree once the OTUs were scattered in all the clusters.

The multiple sequence alignments of protein families provide not only phylogenetic information but can also be used to detect functional and structurally important positions in a protein by analyzing the context of its homolog family. The presence of heavily conserved positions indicates which residues are susceptible to strong selection pressure in that family and probably necessary for function (e.g., catalytic residues) or structure. Additional information can be obtained by identifying coevolved residue sets, i.e., those that may not be conserved but tend to appear simultaneously in a part of the proteins. These sets may be the result of divergent evolution in a protein family: different subclasses, which may even be present in the same species as paralogs, evolve different residue sets related to different functions, and this can be assessed by detecting residue correlations in a multiple sequence alignment.

In this study, a multiple sequence alignment analysis of the ArsC domain PF03960 showed that the catalytic cysteine (Cys12) is not conserved across all homologs. This result is easily explained by the fact that this protein family includes not only arsenate reductase but other proteins that do not need such a Cys; even in other proteins containing Cys12, the Cys may have a different function other than being the binding site for AsV, as is the case of the Spx proteins. They tend to possess the coevolved set 3 (Cys12-Leu22), but in this case, the catalytic cysteine works as a disulfide stress sensor.

The amplicons of ArsCs obtained in this study encompassed a region rich in putatively class-defining positions, even though they lack the Cys12. The highly conserved residues, together with the coevolved sets found in the sequences amplified in this study, strongly suggest that they are indeed capable of performing AsV reduction: all the sequences possessed the coevolved set 1, and none of them possessed coevolved set 4 (discarding the paralogs YffB and YusI). Furthermore, the low genetic variation in positions required for AsV reduction in our sequences suggests a strong selective pressure to maintain the gene’s functionality^60^.

Regarding *aioA* sequences, although the molybdopterin domain also seems to contain many positions that show different prevalences among the many classes annotated at SwissProt (AIOAs, DMSAs, NDUS1s, NARGs, NAPAs, NUOGs, FDHLs, TORAs, TORZs and others with fewer members), very few of them lie in the region amplified in this work. Therefore, the conserved residues found in our AioA sequences were insufficient for detecting any putative function. Moreover, the binding site annotated for the two AioAs in SwissProt (for the *A*. *faecalis* AioA, it would be His196, Glu204, Arg420 and His424) is unfortunately very poorly populated among proteins with molybdopterin-like domains (even the most prevalent residue, Glu228, is present in only 9% of the sequences). Hence, identification of putative AioAs needs to be based on similarity comparison to other proteins.

In conclusion, our results show that a long-term As-contaminated tropical freshwater sediment contains a highly diverse microbiota. Our findings highlighted the importance of As-ECs, which originated from sediment, for identifying As-transforming bacteria. Such strategy revealed, in a single study, a plethora of taxa performing As oxidation and reduction, most of which are described here for the first time. The *aioA*, *arsC* and *arrA* phylogenies suggested the occurrence of horizontal gene transfer and showed a great sequence diversity, particularly in the case of *arrA*; these genes clearly segregated according to the conditions of their ECs, suggesting they may be novel functional variants. Bioinformatic analyses of ArsC’s and AioA’s homolog families identified highly conserved amino acid residues and coevolved sets that are effective tools for discriminating familial subgroups and for assessing the proteins’ functional and structural integrities. Our findings indicate that ArsCs from studied environment are functional since they conserve all amino acids required for AsV resistance, which also suggests they are under strong selective pressure. Importantly, these findings highlight the role of bacteria in As mobility, mainly in regard to the untapped diversity of DARPs and ARMs increasing their toxicity to the aquatic biota. Future studies should focus on bioprospecting environmentally friendly bacteria and quantifying As-related genes to identify whether As oxidation or reduction is more frequent in natural environments.

## Methods

### Study area and sample collection

Sediment samples were collected from the Mina Stream (19°58′46.80″S–43° 49′17.07 W), located in the Brazilian gold mining area known as the Iron Quadrangle (Minas Gerais state), one of the world’s largest mining regions. This stream has suffered varying degrees of stress due to metal pollution (e.g., As), exceeding the maximum allowable concentrations established by Brazilian environmental regulations (CONAMA)^17^. The characteristics of the Iron Quadrangle have been described in detail by Costa *et al*.^16^.

The sediment samples were aseptically taken at a depth of 30 cm in order to avoid the oxygen-richer area that exists from a few millimeters to 15 cm in depth^63, 64^. The samples were taken at three points that were 1 m distant from each other during the dry season of 2013. The three samples, were pooled together and kept on ice during transport to the laboratory, where they were further processed.

Physiochemical characteristics such as temperature, pH and dissolved oxygen were measured *in situ* with a Horiba multiprobe, model U-22^63^. Total nitrogen, ammonium, nitrite, nitrate, and total and soluble reactive phosphorus were measured as previously described^65, 66^. Metal and metalloid concentrations of the water and sediment samples were determined using an inductively coupled plasma-optical emission spectrometer (ICP-OES, Optima 7300 DV, PerkinElmer).

### Arsenic enrichment cultures

The enrichment of anaerobic AsIII- and AsV-resistant bacteria was carried out in anaerobic jars containing 100 mL of CDM medium (0.012 mM Fe_2_SO_4_, 7 mM Na_2_SO_4_, 0.0574 mM K_2_HPO_4_, 9.5 mM NaHCO_3_, 18.7 mM NH_4_Cl, 8.12 mM MgSO_4_, 0.457 mM CaCl_2_ and 44.6 mM sodium lactate (as the organic carbon source), pH 7.2) supplemented with 2 mM sodium arsenite or 10 mM sodium arsenate. Both jars were inoculated with 10 g of sediment and incubated at 28 °C for seven days. After this period, the genomic DNA was extracted and purified.

### DNA extraction

The metagenomic DNA from 10 g (wet weight) of sediment and the genomic DNA from AsIII- and AsV-enrichment cultures were extracted and purified using the PowerSoil DNA Extraction Kit (MoBio Laboratories, USA) according to the manufacturer’s instructions. The total DNA was quantified by measuring the absorbance of the sample at 260 nm using a NanoDrop 2000 spectrophotometer (Thermo Fisher Scientific, USA), while the quality was determined by measuring the absorbance at 230, 260 and 280 nm and calculating the 260/280 and 260/230 ratios. The DNA was stored at −20 °C until further processing.

### Construction of *arsC*, *arrA* and *aioA* genes libraries

The PCR screens targeting the *arsC*, *arrA* and *aioA* genes of AsIII-EC, AsV-EC and sediment were obtained as previously described by Sun *et al*.^67^, Malasarn *et al*.^68^ and Hamamura *et al*.^69^, respectively. Information about the primers and amplification conditions used in this study are summarized in Supplementary Table S2. The examined *arsC* gene was similar to the glutaredoxin-dependent arsenate reductase enzyme from the *Escherichia coli* R773 plasmid^70^. The amplicons of the *arsC*, *arrA*, and *aioA* genes were gel-purified using the Silica Bead DNA Gel Extraction Kit (Fermentas, Canada). The PCR products were cloned into the vector pJET1.2/blunt (Fermentas, Canada) and propagated in *Escherichia coli* XL1-Blue (Phoneutria, Brazil) electrocompetent cells according to the manufacturer’s instructions.

### Sequencing and phylogenetic analysis

The *arsC*, *arrA*, and *aioA* gene sequences were obtained with an ABI Prism 3130 automatic DNA sequencer (Applied Biosystems, USA). The vector sequences were removed using the web-based tool VecScreen (http://www.ncbi.nlm.nih.gov/tools/vecscreen/) and sequences were assembled into contigs using the software package Phred/Phrap/Consed (http://www.phrap.org/phredphrapconsed.html). Chimeras were identified using Bellerophon (http://comp-bio.anu.edu.au/bellerophon/bellerophon).

The sequences were aligned in MEGA 6.0^71^ using the ClustalW tool^72^ with its default settings and were manually edited. The OTUs from the *arsC*, *arrA*, and *aioA* gene clone libraries were identified with DOTUR software^73^ using an identity cutoff of 97%. The Good’s libraries coverage were calculated using the equation C = 1 − (n/N) × 100, where n is the number of unique OTUs and N is the number of sequences analyzed in the library^74^. The *arsC*, *arrA*, and *aioA* gene sequences were compared with those available in the GenBank database (http://www.ncbi.nlm.nih.gov/). The BLASTX search tool was used to identify potential homologs for evolutionary analyses.

The obtained *arrA*, *arsC* and *aioA* sequences were further used in phylogenetic analyses. To increase the phylogenetic signal, evolutionary trees were reconstructed by using nucleotides once the amino acid sequences had become so short that they could lead to overparameterization^16^. For each set of genes, the nucleotide sequences that were obtained from the ECs and the sediment, plus the reference sequences retrieved from the GenBank database (http://www.ncbi.nlm.nih.gov/), were aligned using MAFFT 7 with iterative refinement by the G-INS-i strategy^75^ and then manually refined using Jalview^76^. Redundant sequences were filtered using the Decrease Redundancy tool available at ExPaSy (www.expasy.org) with the following parameters: 99% for maximum similarity and 30% for minimum similarity. Identical sequences were clustered as single OTUs. For the construction of the phylogenetic trees, the maximum likelihood method (ML), as implemented in PhyML, was used and is available through Phylemon2 (http://phylemon.bioinfo.cipf.es/)^77^. Distinct evolutionary models were also tested with the PhyML Best AIC tree option, and support values for each node were estimated using the Akaike Likelihood Ratio Test (aLRT). The Akaike Information Criterion (AIC) compares the likelihood of tested models to determine which one best fits the data. The trees were visualized and edited using the FigTree software (tree.bio.ed.ac.uk/software/figtree). Accession numbers of reference sequences used for phylogenetic reconstruction are summarized in Supplementary Table S3.

### 16S rRNA gene library preparation and sequencing

The Illumina MiSeq platform was used to generate the taxonomic profiles of the bacterial community. The 16S rRNA V3-V4 region was amplified by PCR using primers described by Klindworth *et al*.^78^, and amplicon libraries were prepared according to the manufacturer’s protocol (http://support.illumina.com/documents/documentation/chemistry_documentation/16s/16s-metagenomic-library-prep-guide-15044223-b.pdf).

### Data processing and statistical analyses

The raw reads generated from MiSeq were processed using Mothur software v.1.36.1^79^, following the MiSeq SOP pipeline^80^. Reads of low quality (Q ≤ 20, with more than two ambiguities and eight homopolymers) or outside the range of 400–428 bp were discarded. The reads were then preclustered, and chimeras were identified and removed using the Uchime method. The contigs were aligned and classified against the Greengenes August 2013 release of the 16S rRNA database^81^, using the Bayesian classifier while requiring an 80% pseudobootstrap confidence score^82^. Archaea, Eukaryota, chloroplasts, mitochondria and unknown reads were discarded. The remaining high-quality reads were then clustered into OTUs based on 97% genetic similarity using the average-neighbor method.

The statistical analyses were performed with R software^83^ using the Phyloseq package^84^. Singletons were removed from the dataset beforehand. For alpha and beta diversity analyses, all samples were normalized to the lowest number of reads with the “rarefy_even_depth” Phyloseq command. Alpha diversity was calculated using the ACE and Chao1 species estimators and Shannon and inverse Simpson diversity indexes. Beta diversity was assessed with Bray-Curtis distances that were sorted by principal coordinate analysis (PCoA).

### Computational analysis of protein families

Multiple sequence alignments of protein domain families were obtained directly from Pfam^85^. ArsCs are represented in Pfam under entry PF03960. The sequences of AioA-like proteins usually have multiple domains; the conserved region that is used for PCR detection is present in the molybdopterin domain (PF00384). Due to their short length, the ArrA amino acid sequences obtained in this study were not included in this analysis.

The alignments for the conservation and coevolution analysis were performed in PFSTATS, a computer implementation of a previously described methodology to detect functional determinants in protein families^86^. Fragments and highly similar sequences (>70% identity) were removed, resulting in 2239 sequences for ArsCs and 2717 sequences for the molybdopterin domain. To detect the coevolved positions, pairwise residue-specific correlations were calculated using the following procedure — two residue-pairs (e.g., His14 and Cys23) were considered correlated if the presence of one of them increased the frequency of the other by more than 80%, with a p-value smaller than 10^−10^. Furthermore, the two residues must have been present in at least 20% of the sequences, as determined by Dima & Thirumalai^87^. For ArsCs, the sequence numbering refers to *E*. *coli* ArsC (UniProt code P08692), unless noted. For molybdopterin, A0A0U0D0V4 from *Streptococcus pneumoniae* was used as the reference.

The overall distribution of amino acid residues of translated sequences obtained in this study was determined using the WebLogo V.2.8.2 online tool (http://weblogo.berkeley.edu/).

### Data availability

The authors declare that the data generated in this study are deposited in the GenBank database under the accession numbers: PRJNA361507, KX767688-KX767785, KX767619-KX767687, and KX767523-KX767618.

## Electronic supplementary material

Supplementary tables and legends

Dataset 1
