# Supplementary material for: Novel arsenic-transforming bacteria and the diversity of their arsenic-related genes and enzymes arising from arsenic-polluted freshwater sediment
Source: Sci Rep. 2017 Sep 11;7:11231. doi: 10.1038/s41598-017-11548-8 (PMC5593903; doi:10.1038/s41598-017-11548-8)
Supplement: Supplementary file 1 — Supplementary tables and legends [file 41598_2017_11548_MOESM1_ESM.pdf]

Novel arsenic-transforming bacteria and the diversity of their arsenic-related genes and enzymes arising from arsenic-polluted freshwater sediment

Maria L. S. Suhadolnik[1], Ana P. C. Salgado[1], Larissa L. S. Scholte[2], Lucas Bleicher[3], Patrícia S. Costa[1], Mariana P. Reis[1], Marcela F. Dias[1], Marcelo P. Ávila[1], Francisco AR Barbosa[1], Edmar Chartone-Souza[1], Andréa M. A. Nascimento

Supplementary Information

Supplementary Table S1. The 16S rRNA dataset.

Supplementary Table S2. Primers and thermocycling conditions used for clone library construction.

Gene	Primers	Nucleotide sequence 5'-3'	Amplicon	Thermal cycling conditions	Rerefences
aioA	aroA95f<br>aroA599r	TGYCABTWCTGCAIYGYIGG<br>TCDGARTTGTASGCIGGICKRTT	547 nucleotides long, spanning the regions 75-622 of the *Sulfurihydrogenibium yellowstonense's* aioA.	Initial denaturation at 94°C for 5 min; 9 cycles of 94°C for 45 s and 54°C for 45 s (decreased by 0.5°C after each cycle); followed by 1.5 min at 72°C; other 25 cycles of 94°C for 45 s, 50°C for 45 s and 1.5 min at 72°C; and final extension of 7 min at 72°C.	69
arsC	amlt-42-f<br>amlt-376-r	TCGCGTAATACGCTGGAGAT<br>ACTTTCTCGCCGTCTTCCTT	346 nucleotides long, spanning the regions 54 - 400 of *Escherichia coli* plasmid R773' *arsC.*	Initial denaturation 95°C for 3 min, followed by 40 cycles of 95°C for 15 s, 60°C for 15 s and 72°C for 15°C s, and final extension of 72°C for 10 min.	67
arrA	ArrAfwd<br>ArrArev	AAGGTGTATGGAATAAAGCGTTTgtbgghgaytt<br>CCTGTGATTTCAGGTGCCcaytyvggngt	174 nucleotides long, spanning the regions 869-1043 of *Shewanella* strain ANA-3 *arrA* gene.	Initial denaturation at 95°C for 10 min, 40 cycles of 15s at 95°C, 40s at 50°C and 1 min at 72°C, and final extension at 72°C for 10 min.	68

Supplementary Table S3. Accession numbers of reference sequences retrieved from NCBI database.

Gene	Accession number	Taxonomic ID
aioA	EF015461.1	*Ancylobacter* sp. OL1
aioA	EF015462.1	*Hydrogenophaga* sp. CL3
aioA	EF015459.1	*Thiobacillus* sp. S1
aioA	CBK43383.1	*Nitrospira defluvii*
aioA	EF015463.1	*Bosea sp. WAO*
aioA	KC012943.1	Sinorhizobium sp. IK-A2
aioA	EU304278.1	*Aminobacter* sp. 86
aioA	HF570939.1	*Aliihoeflea aestuarii* 2WW
aioA	JX489054.1	Uncultured bacterium clone aroA-riceroot-3
aioA	KC012943.1	*Sinorhizobium* sp. IK-A2
aioA	JX489070.1	Uncultured bacterium clone aroA-riceroot-16
aioA	EF015458.1	*Sinorhizobium* sp. DAO10
aioA	EU304277.1	*Pseudomonas* sp. 46
aioA	EU304268.1	*Acidovorax* sp. 75
aioA	EU304264.1	*Leptothrix* sp. S1-1
aioA	AB638435.1	*Hydrogenophaga defluvii*
aioA	EU304260.1	*Thiomonas arsenivoran* DSM 16361
aioA	GU731381.1	Bacterium enrichment culture clone aoxheteroB90_4W
aioA	HQ449659.1	*Rhodococcus* sp. 46AAIII_aioA
aioA	HQ449657.1	*Flavobacterium* sp. 18AGV
aioA	HQ449648.1	*Bacillus* sp. 21AAIII
aioA	HQ449664.1	*Flavobacterium* sp. 9AAV
aioA	HQ449668.1	*Agromyces* sp. 44AGV
aioA	AB974345.1	*Cupriavidus* sp. iCE102s
aioA	KU950313.1	*Acinetobacter sp.*
aioA	EGV16077.1	*Thiocapsa marina* 5811

aioA	CC032987.1	*Micromonospora sp.*
aioA	AY297781.1	*Alcaligenes faecalis*
aioA	CP007201.1	*Sulfurospirillum multivorans*
aioA	AF509588.1	*Cenibacterium arsenoxidans*
aioA	KX274407.1	*Gemmobacter aquatilis* clone aioA-14
aioA	KX274408.1	*Agrobacterium tumefaciens*
aioA	CAV25302.1	*Vibrio splendidus*
aioA	FLQP01000018.1	*Vibrio atlanticus*
aioA	CTQ69902.1	*Labrenzia alba*
aioA	CP002778.1	*Thermus thermophilus*
aioA	CP001337.1	*Chloroflexus aggregans*
aioA	CP001097.1	*Chlorobium limicola*
aioA	KC777342.1	Uncultured bacterium
aioA	KC777341.1	Uncultured bacterium
aioA	KJ794092.1	Uncultured bacterium
aioA	JX489054.1	Uncultured bacterium
aioA	JX489070.1	Uncultured bacterium
aioA	LC012305.1	Uncultured bacterium
aioA	LC012258.1	Uncultured bacterium
aioA	JX863395.1	Uncultured bacterium
aioA	AB905510.1	Uncultured bacterium
aioA	KR095425.1	Uncultured bacterium
aioA	KP072514.1	Uncultured bacterium
aioA	KF841157.1	Uncultured bacterium
aioA	KX711875	aioA_MS_AsV
aioA	KP072513.1	Uncultured bacterium
aioA	KX711877	aioA_MS_AsIII
arrA	AY660886.1	Shewanella sp.

arrA	AY660885.2	Bacillus arseniciselenatis
arrA	AY283639.1	Bacillus selenitireducens MLS10
arrA	AY660883.1	Chrysiogenes arsenatis
arrA	AY660884.2	Sulfurospirillum_barnesii
arrA	DQ220794.1	Desulfosporosinus sp. Y5
arrA	JF827143	Geobacter uraniireducens
arrA	EU723191.1	Halarsenatibacter silvermanii SLAS-1
arrA	EU186649.1	Bacillaceae bacterium
arrA	AP012547.1	Sulfuritalea hydrogenovorans
arrA	LK996017.1	Desulfitobacterium hafniense
arrA	FN645201.1	Uncultured bacterium_ arrA
arrA	AY707761.1	Uncultured bacterium_ arrA
arrA	AY707760.1	Uncultured bacterium_ arrA
arrA	AY707769.1	Uncultured bacterium_ arrA
arrA	AY707762.1	Uncultured bacterium_ arrA
arrA	FN645211.1	Uncultured bacterium_ arrA
arrA	FN645212.1	Uncultured bacterium_ arrA
arrA	EU723192.1	Uncultured bacterium_ arrA
arrA	AB769395.1	Uncultured bacterium_ arrA
arrA	KP061102.1	Uncultured bacterium_ arrA
arrA	KP061103.1	Uncultured bacterium_ arrA
arrA	JX845272.1	Uncultured bacterium_ arrA
arrA	KP061118.1	Uncultured bacterium_ arrA
arrA	KP061117.1	Uncultured bacterium_ arrA
arrA	KP061116.1	Uncultured bacterium_ arrA
arrA	AB769393.1	Uncultured bacterium_ arrA
arrA	AB769394.1	Uncultured bacterium_ arrA
arrA	KR051570.1	Uncultured bacterium_ arrA
arrA	FJ747610.1	Uncultured bacterium_ arrA
arrA	DQ155370.1	Uncultured bacterium_ arrA

arrA	DQ155371.1	Uncultured bacterium_ arrA
arrA	JX845270.1	Uncultured bacterium_ arrA
arrA	JX845272.1	Uncultured bacterium_ arrA
arrA	JX845271.1	Uncultured bacterium_ arrA
arrA	KP061104.1	Uncultured bacterium_ arrA
arsC	DQ398936.1	Sinorhizobium meliloti
arsC	JN609521.1	Hoeflea sp.
arsC	JN609518.1	Agromyces sp.
arsC	AJ704863.3	Klebsiella pneuminiae
arsC	CP000699.1	Sphingomonas wittichii
arsC	KP060430.1	Uncultured bacterium_arsC
arsC	KP060508.1	Uncultured bacterium_arsC
arsC	JX489121.1	Uncultured bacterium_arsC
arsC	KP060526.1	Uncultured bacterium_arsC
arsC	KP726882.1	Uncultured bacterium_arsC
arsC	KX711835	arsC_MS_AsV
arsC	KX711785	arsC_MS_AsV
arsC	KX711839	arsC_MS_AsV
arsC	KX711795	arsC_MS_AsV
arsC	KX711786	arsC_MS_AsIII
arsC	KX711793	arsC_MS_AsIII
arsC	KP726881.1	Uncultured bacterium_arsC
arsC	JX489106.1	Uncultured bacterium_arsC
